# Identifying novel genetic alterations in pediatric acute lymphoblastic leukemia based on copy number analysis

**DOI:** 10.1186/s13039-020-00491-5

**Published:** 2020-06-26

**Authors:** Jéssica Almeida Batista-Gomes, Fernando Augusto Rodrigues Mello, Edivaldo Herculano Corrêa de Oliveira, Michel Platini Caldas de Souza, Alayde Vieira Wanderley, Laudreisa da Costa Pantoja, Ney Pereira Carneiro dos Santos, Bruna Cláudia Meireles Khayat, André Salim Khayat

**Affiliations:** 1 Oncology Research Center, Federal University of Pará, Belém, Brazil; 2 Cell culture and Cytogenetic Laboratory, Evandro Chagas Institute, Ananindeua, Brazil; 3 Octávio Lobo Children’s Cancer Hospital, Belém, Brazil

**Keywords:** ALL (acute lymphoblastic leukemia) childhood leukemia, *DMBT1*, *KIAA0125*, *PRDM16*, Copy number variations

## Abstract

Copy number variations (CNVs) analysis may reveal molecular biomarkers and provide information on the pathogenesis of acute lymphoblastic leukemia (ALL). We investigated the gene copy number in childhood ALL by microarray and select three new recurrent CNVs to evaluate by real-time PCR assay: *DMBT1*, *KIAA0125* and *PRDM16* were selected due to high frequency of CNVs in ALL samples and based on their potential biological functions in carcinogenesis described in the literature. *DBMT1* deletion was associated with patients with chromosomal translocations and is a potential tumor suppressor; *KIAA0125* and *PRDM16* may act as an oncogene despite having a paradoxical behavior in carcinogenesis. This study reinforces that microarrays/aCGH is it is a powerful tool for detection of genomic aberrations, which may be used in the risk stratification.

## Introduction

Acute lymphoblastic leukemia (ALL) is the most common cancer in children [1]. Leukemia represents the ninth most common cancer in Brazil and the fifth most frequent in the north region [2]. Advances in cytogenetics and molecular cytogenetics has allowed the identification of genetic aberrations in more than 80% of ALL cases [3]. Establishing genetic background in ALL patients is important for the diagnosis, risk classification and therapeutic interventions [3]. However, some patients do not have an established chromosomal aberration, which complicates the risk classification.

Recent analysis has shown that copy number variations (CNVs) are common in ALL and leukemia in general, especially in genes involved in transcription, cell cycle regulation and B-cell differentiation, (e.g., *CDKN2A/B*, *IKZF1*, *ETV6*, *EBF1*, *PAX5*, *BTG1* and *PAR1*) [4]. Additional CNVs could be helpful to refine ALL prognostic. The prognostic effect of CNVs depends on the other factors, such as the presence of additional molecular or cytogenetic aberrations; this situation reinforces the need to analyze these combined alterations [5].

The aim of this report is to assess and evaluate CNVs identified by aCGH from a cohort of Brazilian children with ALL. Three new recurrent CNVs were further evaluated by qPCR. We highlight that *DMBT1*, *KIAA0125* and *PRDM16* were chosen due to high frequency of aberrations in ALL samples and based on their biological functions as well the data present in the literature.

## Methods

### Patients

A total of 16 ALL pediatric patients (5 ± 3 years) treated at Octávio Lobo Children’s Cancer Hospital were selected for aCGH analysis. Additional 84 ALL pediatric samples were used as validation group in copy number qPCR assay. These patients were classified by immunophenotyping and morphology. Gene fusions were investigated by reverse transcription polymerase chain reaction (RTq-PCR) (Tables 1 and 2). The samples were collected before cancer treatment between 2017 and 2019.

**Table 1:** Characteristics of pediatric patients with ALL included in the study

Characteristic	aCGH(*n* = 16)	qPCR (*n* = 84)
Male: female	08:08	49:35
Median age (y)	6.5	7.4
Median WBC count (× 10^9^/L)	73	69.4
Immunophenotype		
B	15	80
T	1	4
Chromosomal alteration		
*TCF3-PBX1* (n)	6	15
*BCR-ABL1* (n)	1	9
*MLL-AF4* (n)		5
*ETV6-RUNX1* (n)		7
*SIL-TAL1*(n)		3
NCI risk		
High (n)	7	30
Standard (n)	9	54

**Table 2:** Nucleotide sequence of RTq-PCR primers

Genes	Primers (5′-3′)	Size (bp)	Position	Exons
*TCF3*	CTACTCCCCGGATCACTCAA	20	1086–1105	13
*PBX1*	AGGCTTCATTCTGTGGCAGT	20	3893–3912	2
*MLL*	CGCCCAAGTATCCCTGTAAA	20	4071–4090	8
*AF4*	GAGCATGGATGACGTTCCTT	20	1546–1565	8
*BCR*	TCGCAGAACTCGCAACAGT	19	1707–1725	1
*ABL*	ACACCATTCCCCATTGTGAT	20	284–303	3
*ETV6*	TCTCTCATCGGGAAGACCTG	20	1191–1210	5
*RUNX1*	TGCGGTAGCATTTCTCAGC	19	619–637	5
*SIL*	TCCTACCCTGCAAACAGACC	20	73–92	1
*TAL1*	AGGCGGAGGATCTCATTCTT	20	1250–1269	4

The age at diagnosis and white blood cell (WBC) count were the criteria for assigning prognostic risk of ALL, according to the National Cancer Institute (NCI): 1) high risk, WBC count greater than 50 × 10^9^ cells/μL, age 1 year or less, or age 10 years or more; and 2) standard risk, WBC count 50 × 10^9^ cells/μL or less, or between 1 and 10 years of age. The patients with *BCR-ABL1 or MLL-AF4* also were assigned to the NCI high risk group. Written consent forms were obtained from all parents of patients. This study was approved by hospital ethics committee (CAAE: 00905812.1.0000.00.18).

### Array comparative genomic hybridization

Genomic DNA was extracted from peripheral blood by Pure Link Genomic DNA Mini Kit (Invitrogen, California, USA). aCGH was performed using Agilent 4x180k CGH + SNP microarray (Santa Clara, USA). After DNA extraction, a restriction enzyme digestion step and labeling with fluorochrome cyanine 5 were performed using random primers and exo-Klenow fragment DNA polymerase. DNA control was labeled with fluorochrome cyanine 3. DNA samples from the patient and control were combined and hybridized on the microarray. Data were analyzed using the software Agilent’s CytoGenomics v5.0.

### Real-time quantitative PCR

TaqMan Copy Number Assay (Applied Biosystems, California, USA) was used to assess copy number for *DMBT1*, *KIAA0125* and *PRDM16*. Briefly, 1 μL of 10 ng DNA was added to 5 μL of TaqMan Universal Master Mix no UNG, with 0.5 μL of each probe and 3 μL of ultra-pure water. RNase P was used as a control. The amplification protocol consisted of: denaturation at 95 °C for 10 min, followed by 40 cycles of 95 °C for 15 s and 60 °C for 1 min. Relative quantification was determined using the 7500 Real-time PCR system and all samples were analyzed in quadruplicate. After amplification, we imported the experiment results containing threshold-cycle values for the copy number and reference assay into the Copy Caller Software v2.0 for post-PCR data analysis as previously described [6].

### Statistical analysis

Fisher’s exact test was used to compare the distribution of aberrations between subgroups (high or standard risk; positive or negative for chromosomal translocation) and pathological features of the patients; Odds ratio (OR) with a 95% confidence interval (CI) were also calculated through the statistical program BioEstat® v5.0 [7]. *p*-values less than 0.05 were considered significant.

## Results

### aCGH profiling identifies recurrent alterations

aCGH date were available for all 16 cases, the average of copy number variations (CNVs) was 8.3 per sample. Gains were the most frequent event, the most frequently gained regions were on chromosomes 14 (q32.33) and 10 (q26.13), these regions include *KIAA0125* (a lncRNA) and *DMBT1* genes, respectively. Frequent losses were identified on chromosome 7 (7p12.2) and 9 (p21.3), which includes *IKZF1* and *CDKN2A/B* genes, respectively.

Recurrent CNVs included gains of cytobands 14q (93.75%), 2p (68.75%), 17q (62.5%), 9q (56.25%), 10q (56.25%), 19q (56.25%), 22q (56.25%), 1p (50%), 7q (50%), 8p (50%) and 21q (50%); losses involving 7p (62.5%), 9p (56.25%), 15q (47.75%), 4q (37.5%) and 12q (31.25%). The list of recurrent CNVs found in at least two samples is provided in Table 3.

**Table 3:** The most frequent copy number variations found in pediatric ALL by aCGH

Frequency % (n = 16)	Chromosome	Reference region	Variant type	Genes involved
94	14	q32.33	Amp	*KIAA0125*^*a*^
75	14	q11.22	Del	Several genes
62.5	7	7p12.2	Del	*IKZF1*
56.25	9	p21.3	Del	*CDKN2A/B,MTAP*
56.25	10	q26.13	Amp	*DMBT1*^*a*^
56.25	22	q11.22	Amp	*MIR650, IGLL5*
50	15	q11.1	Del	*HERC2P3*
50	1	p36.32	Amp	*PRDM16*^*a*^
50	19	q13.32	Amp	*KLC3, ERCC2*
37.5	4	q13.2-q13.3	Del	*UGT2B4*
31.25	12	q21.33-q22	Del	*BTG1*
25	13	q14.2	Del	*RB1*
19	7	p14.1	Del	*TRGC2, TARP*
19	11	q23.3	Del	*KMT2A*
19	12	12p13	Del	*ETV6*
12.5	7	p21.3-p15.2	Del	Several genes
12.5	3	q29	Del	*DLG1*
6.25	21	iAMP21	Amp	*RUNX1*

^*a*^Alterations have never been described in literature for ALL. *Amp* amplification. *Del* deletion

All patients have alteration in at least one of the main genes associated with ALL; *ETV6*, *RUNX1*, *IKZF1*, *KMT2A (MLL)* and *BTG1* (Table 3.). The median of alterations in standard (SR) and high risk (HR) group were 56.6 (±15.4) and e 52.2 (±14.2), respectively. We confirmed the association of CDKN2A/*B* losses with positive cases for *TCF3-PBX1* or *BCR-ABL1* (*p* < 0.05). There was no statistically significant difference in the number of CNVs between patients with (CT+) or without (CT-) chromosomal translocation.

### CNV evaluation by real-time qPCR

**Fig. 1:**
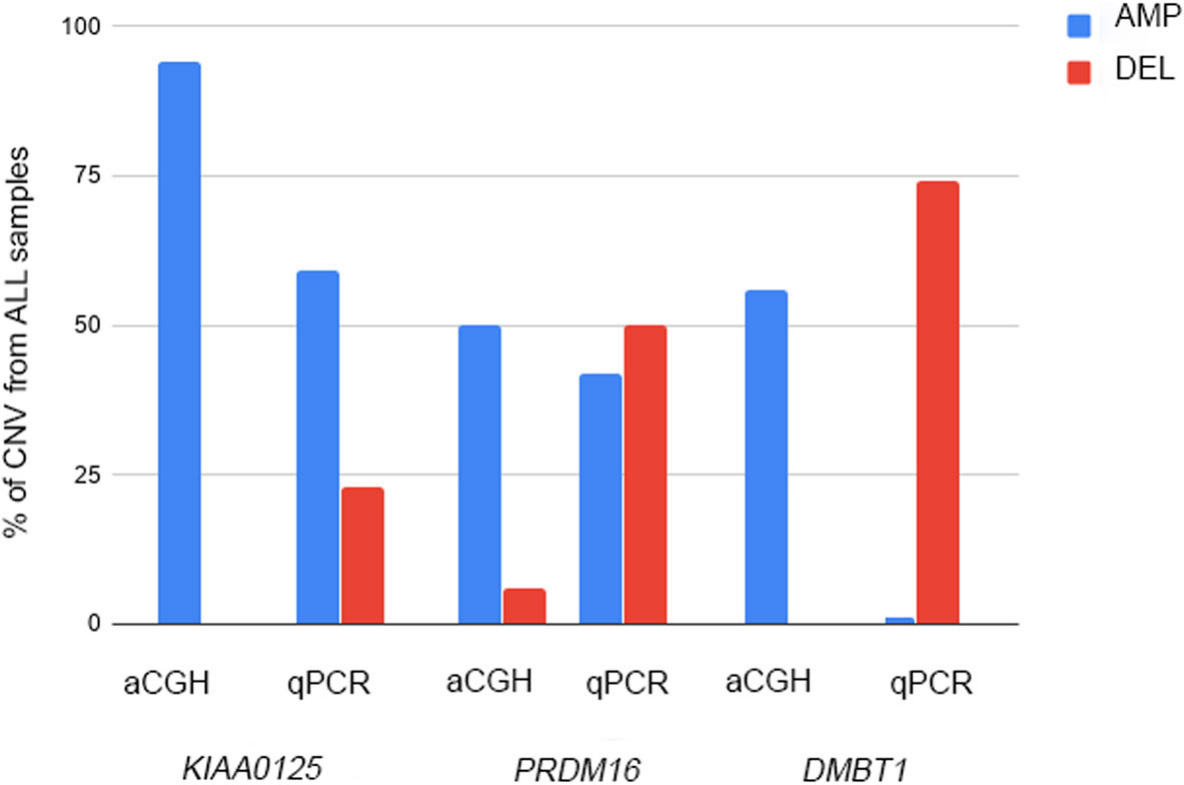
Frequency of copy number variation of *PRDM16*, *KIAA0125* and *DMBT1* identified by aCGH and qPCR. aCGH for 16 samples; qPCR for 48 samples. AMP: amplification; DEL: deletion

To validate aCGH results *DMBT1*, *KIAA0125* and *PRDM16* genes were analyzed by qPCR. Genes were chosen due to the high frequency of aberrations found in samples and based on their biological function (mainly transcriptional regulation) described in literature. It is noted that the CNV found in these genes are described here for the first time in leukemia, especially in ALL. The aberrations of the three selected genes identified from aCGH and qPCR were illustrated in Fig. 1.

**Fig. 2:**
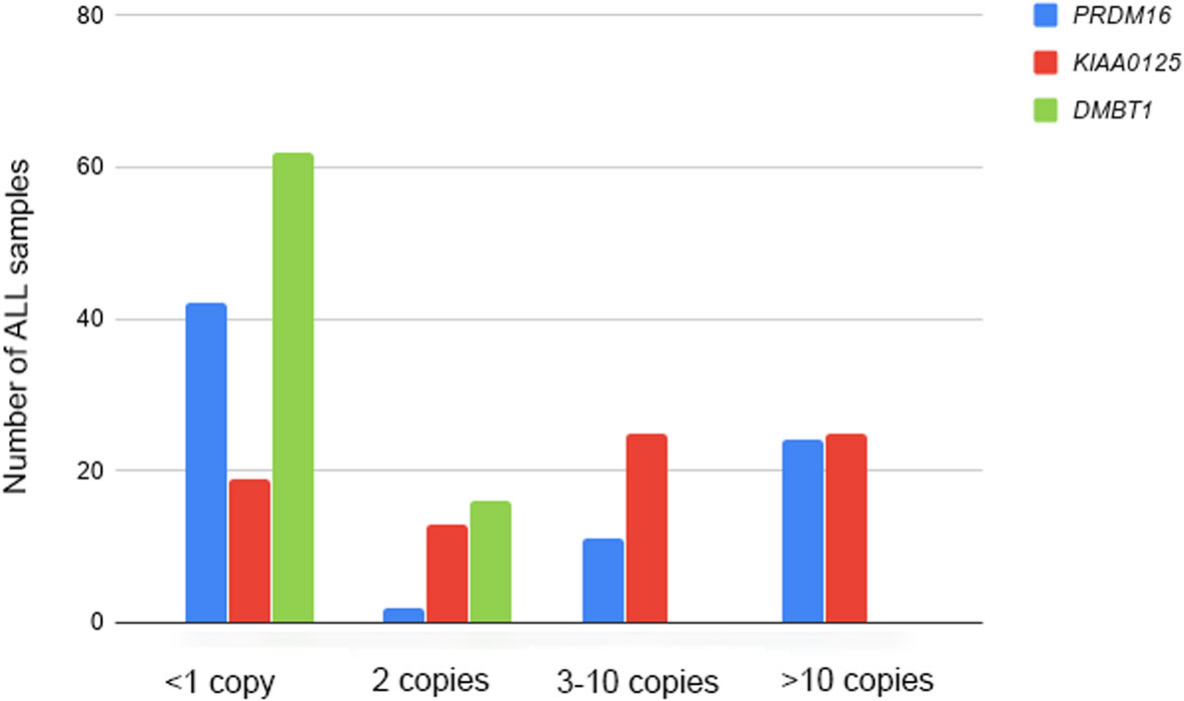
Copy number of *PRDM16*, *KIAA0125* and *DMBT1* in ALL samples identified by qPCR

The results of qPCR were compared between positive (CT+) or negative (CT-) for gene fusions subgroups. *DMBT1* deletion was observed in 74% of patients (*n* = 62; 97.4% of CT+ and 53% of CT-); *KIAA0125* amplification was detected in 59% of cases (*n* = 50; 95% of CT+ and 29% of CT-), these amplifications were more frequent in cases CT+; while *PRDM16* was deleted in 50% of patients (*n* = 42; 87% CT- and 8% CT+), amplifications were observed in 42% of samples, which only correspond to CT+ cases (Fig. 2).

Curiously, 50% (25/50) and 69% (24/35) of *KIAA0125* and *PRDM16* amplifications, respectively, were high-level amplifications (> 10 copies), however, classification of cases according to the level of amplification did not result in any significant association.

The frequency of aberrations in *DMBT1*, *KIAA0125* and *PRDM16* according to NCI risk group, gender, age and cytogenetic findings are show in Tables 4 and 5 Statistical analysis showed that *DMBT1* deletion was more common in patients with > 1 to ≤10 years (OR = 3.38; 95% IC = 1.15–9.89) and more common in NCI-SR cases (OR = 0.198; 95% IC = 0.07–056). *DMBT1* deletion also was associated with CT+ samples (OR = 33.2; 95% IC = 4.19–263.55) (Tables 4 and 5).

**Table 4:** Frequency of alterations according to characteristics of patients

	*DMBT1* deletion		*KIAA0125* amplification		*PRDM16* deletion	
	Present	Ausent	Present	Absent	Present	Absent
NCI-HR	16	14	19	11	41	13
NCI-SR	46	8	31	23	1	29
p-value	0.0036*		0.6483		*p* < 0.001*	
≤1 years	5	2	4	3	3	4
> 1 to ≤10 years	44	10	34	20	26	28
> 10 years	13	10	12	11	13	10
p-value 1^1^	0.6131		0.9994		1	
p-value 2^2^	0.6693		1		0.6746	
*p*-value 3^3^	0.0433*		0.4497		0.6200	
WBC > 50	16	5	16	5	8	13
WBC ≤ 50	46	17	34	29	34	29
p-value	1		0.0802		0.4568	
Leucopenia	5	0	5	0	0	5
Leucocytosis	54	18	40	32	37	35
p-value	0.3336		0.0720		0.0554	
Male	38	11	31	18	21	28
Female	24	11	19	16	21	14
p-value	0.4515		0.5002		0.1839	

*NCI-HR* NCI-High risk; *NCI-SR* NCI Standard risk; *WBC* White blood count. ^1^ ≤ 1 years versus > 1 to ≤10 years; ^2^ ≤ 1 years versus > 10 years; ^3^ > 1 to ≤10 years versus > 10 years. *Significant difference between groups with and without aberrations, *p* ≤ 0.05, Fisher’s exact test

**Table 5:** Frequency of alterations according to cytogenetic subgroups

	*DMBT1* deletion		*KIAA0125* amplification		*PRDM16* deletion	
	Present	Ausent	Present	Absent	Present	Absent
*BCR-ABL1*	9	0	9	0	1	8
Absence	53	22	41	34	41	34
p-value	0.1036		0.0032*		0.0294*	
*ETV6-RUNX1*	7	0	7	0	0	7
Absence	55	22	43	34	42	35
p-value	0.1817		0.0381*		0.0119*	
*MLL-AF4*	5	0	4	1	1	4
Absence	57	22	46	33	41	38
p-value	0.3195		0.6438		0.3597	
*TCF3-PBX1*	14	1	14	1	1	14
Absence	48	21	36	33	41	28
p-value	0.0625		0.00031*		0.0003*	
*SIL-TAL1*	3	0	3	0		
Absence	59	22	47	34	0	3
p-value	0.5634		0.2685		42	39
					0.2410	
CT+	38	1	37	2	3	36
CT-	24	21	13	32	39	6
p-value	p < 0.001*		p < 0.001*		p < 0.001*	

*CT+* chromosome translocation positive; *CT* chromosome translocation negative. *Significant difference between groups with and without aberrations, *p* ≤ 0.05, Fisher’s exact test

*KIAA0125* amplification was associated with CT+ cases (OR = 45.5; 95% IC = 9.54–217-16). *PRDM16* gene deletion was associated with NCI-HR patients (OR = 91.4; 95% IC = 11.32–738.6) and CT- cases (OR = 0.01; 95% IC = 0.00–0.05) (Tables 4 and 5), while amplification was related to CT+ samples (*p*-value = < 0.001), data not shown in Tables 4 and 5).

## Discussion

All patients analyzed by aCGH showed a heterogeneous copy-number pattern. We identified 133 CNVs, 18 them involved the most frequent changes already known or not yet related to ALL (Table 3). Unlike previous studies, here, amplifications were more frequent than deletions, possibly due the small sample number and the presence of hyperdiploid cases. On the other hand, similar to antecedent studies [4, 8, 9], the more frequently altered genes were related to cell cycle regulation (*ETV6*), tumor suppression (*CDKN2A/B*), apoptosis regulation (*BTG1*) and others (Table 3).

In agreement with the literature, in our study deletions of *CDKN2A/B* were associated with positive cases for *TCF3-PBX1* or *BCR-ABL1. CDKN2A/B* are tumor suppressor genes acting in cell growth regulation and apoptosis [10]. The deletion of these genes are associated with poor prognosis, high white blood cell count and older age at diagnosis and *BCR-ABL1* or *TCF3-PBX1* translocations [11–13]; all characteristics found in our study group.

The aCGH study also identified for the first-time recurrent alterations of *DMBT1*, *KIAA0125* and *PRDM16* in ALL (Table 3). These genes were verified by qPCR in a larger sample number.

High amplification frequencies observed in aCGH was confirmed by qPCR just for *KIAA0125*. For the *DMBT1* and *PRDM16* deletions were prevalent in qPCR assays. This divergence is probably due to differences in sample size and by the presence of trisomy of chromosomes 1 and 10 in cases with copy number variation in *PRDM16* and *DMBT1*, respectively. But new significant associations were observed for the three genes.

The high frequency of *DMBT1* deletions observed here support aCGH results*. DMBT1* encoding protein belongs to the scavenger receptor cysteine rich (SRCR) super family involved in mucosal immune defense, epithelial differentiation and tumor suppression [14, 15]. Many studies have showed that *DMBT1* deletion or inactivation lead to tumorigenesis by regulating infiltration and metastasis of tumor cells [16]. Altered expression in certain stages of carcinogenesis was identified in different tumor types [17–19]. We found *DMBT1* deletion associated with standard risk and CT+ cases. It is possible that *DMBT1* deletion have a more specific function in development of ALL cases without a high risk chromosomal abnormality (which are mostly classified as standard risk), since only 14% of CT+ cases have high risk biomarker (*BCR-ABL1* or *MLL-AF4*). Thus, *DMBT1* loss collaborate as a secondary event in the progression of disease in CT+ patients, since it is know that chromosomal translocations are primary aberrations [13]. Although *DMBT1* absence is considered a malignancy marker in many epithelial cancers, we reported for the first time *DMBT1* deletion in ALL and we suggest that *DMBT1* may be also involved in hematologic malignancies development.

LncRNAs are involved in gene expression at epigenetic, transcriptional and post-transcriptional level and are considered a strong promise as a biomarker and therapeutic target [20]. In this study, we found that *KIAA0125* amplifications were more common in CT+ patients while in CT- cases, deletions were more prevalent. Recurrent *KIAA0125* amplifications were statistically associated with CT+ cases. CNV or abnormal expression of *KIAA0125* were observed in many tumor types [21–26]. Several recent studies in lncRNAs have shown that they have a critical role in different cancers acting as an oncogene or suppressor, in this sense, the role of *KIAA0125* in carcinogenesis may be cell-type dependent [27]. In colon cancer development, *KIAA0125* may contribute via the regulation of *BCL2* expression by sponging hsa-miR-29b-3p or regulating PI3K-Akt signaling [28]. In addition, Forero-Castro et al. [4] identified losses on 14q32.33 (where *KIAA0125* is located) related to overall survival of children ALL with leukocytosis. In 14q32 there are miRNA clusters that may influence the genes expression levels involved in lymphoid B-cell transformation and differentiation, suggesting that 14q32 losses could be used as a diagnostic marker [4, 29]. Hornung R. et al. (2018) have recently shown that *KIAA0125* could play a mediating role in the influence *RUNX*1 gene fusions have on survival of LMA [30].

It is also presumable that *KIAA0125* may act as miRNA sponges regulates mRNAs expression levels also in ALL, however the exact mechanism of action and possible target genes need to be further investigated. These findings along with our data leading to the assumption that *KIAA0125* plays important role in development of leukemia and reinforce previous studies that suggested that lncRNAs may be utilized as diagnostic and prognostic markers in leukemia [20].

*PRDM16* is characterized by the combination of a conserved N-terminal PR domain and a variable number of zinc fingers [31], it encodes a SMAD binding protein that may repress SMAD-mediated transcription, also functions as a modulator of TGF-beta signaling and exhibit methyltransferase activity [32, 33]. *PRDM16* is involved in various biological processes including maintenance of brown adipocytes and hematopoiesis [34, 35]. Two main PRDM16 isoforms are the full-length and the PR-lacking generated by alternative splicing or alternative use of different promoters [36, 37]. Notably, PRDM proteins sometimes exert opposing effects on tumor development [38, 39]*.*

In the present study most cases have *PRMD16* deletions (50%), however in 90% of CT+ patients this gene is highly amplified (21 samples with > 10 copies) and significantly related to presence of gene fusions. Overexpression of *PRDM16* in AML is associated with worse overall survival [39, 40] and is considered a risk factor for primary induction failure [41]. In addition it is associated with other gene fusions not investigated here [42]. Hu et al. [43] reported that *PRDM16* transforming megakaryocyte-erythroid progenitors into myeloid leukemia stem cells. In another study, *PRDM16* knockdown induced cell proliferation in rhabdoid tumor cells [44], suggesting that *PRDM16* may be an oncogene in leukemia development, although in other tumor types *PRDM16* has a controversial role [45, 46]. Thus, the role of *PRDM16* in cancer biology has been poorly studied and remains to be fully elucidated.

A limitation of this study was the small sample size. However, this is one of the few studies from the northern region of Brazil with genomic analysis in leukemia. This region has a large territorial extension, which makes the diagnosis of cancer a challenge due to its financial viability and the difficult access to geographically isolated regions of cancer treatment centers [47].

In conclusion, this study reinforces that aCGH it is a powerful tool for to identify regions of copy number variations in childhood ALL patients and to identify new genes associated to leukemia. Through this technique, we identified recurrent alterations in genes *DMBT1*, *KIAA0125* and *PRDM16*; these alterations were verified by qPCR and confirmed the possible involvement of these genes in the development of leukemia, especially in ALL. *DMBT1* probably is also a tumor suppressor in leukemia and is associated with standard risk and cases with gene fusions. Although both have a paradoxical behavior in tumorigenesis our data indicates that *KIAA0125* and *PRDM16* may act as oncogene, once amplifications in these two genes were related to gene fusions and leukocytosis, respectively. The combination of two molecular cytogenetics techniques has identified three genes that may be targets for further biological analysis of acute lymphoblastic leukemia.

## Data Availability

The data will not be shared because some analyses of another genes study by aCGH are still being analyzed together with other data and have not yet been published.

## Abbreviations

aCGH: Array Comparative Genomic Hybridization
ALL: Acute lymphoblastic leukemia
CNV: Copy Number Variation
CT+: Chromosome translocation positive
CT-: Chromosome translocation negative
HR: High Risk
NCI: National Cancer Institute
qPCR: quantitative Polymerase Chain Reaction
RT-PCR: Reverse Transcription Polymerase Chain Reaction
SR: Standard Risk
WBC: White Blood Count
